# Diagnostic Value of Immune-Related Genes in Kawasaki Disease

**DOI:** 10.3389/fgene.2021.763496

**Published:** 2021-12-10

**Authors:** Dong Liu, Meixuan Song, Fengchuan Jing, Bin Liu, Qijian Yi

**Affiliations:** ^1^ Department of Cardiovascular Medicine, Ministry of Education Key Laboratory of Child Development and Disorders, National Clinical Research Center for Child Health and Disorders, China International Science and Technology Cooperation Base of Child Development and Critical Disorders, Children’s Hospital of Chongqing Medical University, Chongqing, China; ^2^ Department of Pediatrics, Sichuan Clinical Research Center for Birth Defects, The Affliated Hospital of Southwest Medical University, Luzhou, China; ^3^ Chongqing Key Laboratory of Pediatrics, Chongqing, China; ^4^ Department of Gastrointestinal Surgery, The Affliated Hospital of Southwest Medical University, Luzhou, China

**Keywords:** Kawasaki disease, immune-related genes, immune cells, diagnostic, CIBERSORTx

## Abstract

Kawasaki disease (KD) is a systemic vasculitis that predominantly damages medium- and small-sized vessels, and mainly causes coronary artery lesions (CALs). The diagnostic criterion of KD mainly depends on clinical features, so children could be easily misdiagnosed and could suffer from CALs. Through analysis, a total of 14 immune-related DEGs were obtained, of which *IL1B*, *ADM*, *PDGFC*, and *TGFA* were identified as diagnostic markers of KD. Compared with the non-KD group, KD patients contained a higher proportion of naive B cells, activated memory CD4 T cells, gamma delta T cells, and neutrophils, while the proportions of memory B cells, CD8 T cells, activated memory CD4 T cells, and activated NK cells were relatively lower. In conclusion, immune-related genes can be used as diagnostic markers of KD, and the difference in immune cells between KD and non-KD might provide new insight into understanding the pathogenesis of KD.

## Introduction

Kawasaki disease (KD) is a systemic vasculitis that predominantly damages medium- and small-sized vessels, and mainly causes coronary artery lesions (CALs). KD has become the main cause of acquired heart disease in children; 80% of these cases occur in children aged 6 months to 5 years (Makino et al., 2015), and timely diagnosis and treatment with intravenous immunoglobulin (IVIG) have reduced CALs by about 84% (McCrindle et al., 2017). However, some patients, especially infants under 6 months of age and adolescents, did not meet the criterion of KD, called “incomplete KD”, and it is difficult to make a diagnosis in the early stage of the disease. If there is a special index for KD, the early diagnosis will be easy. On the other hand, delayed diagnosis or missed diagnosis may also cause excessive drug treatment or invasive procedures in KD children. Therefore, an efficient diagnostic index of KD is pursued.

Diagnosis guidelines of KD provided by AHA and the Japanese Ministry of Health are mainly based on clinical symptoms and signs (Ayusawa et al., 2005; McCrindle et al., 2017). Some laboratory data, such as erythrocyte sedimentation rate (ESR), C-reactive protein (CRP), albumin and N-terminal pro-B-type natriuretic peptide (NT-proBNP) level, thrombocytosis, leukocytosis, raised transaminases, hyponatremia, and pyuria, may be helpful in the diagnosis of KD (Cho et al., 2011; Lin et al., 2015; Reddy et al., 2016; Dionne et al., 2017), but all of them are yet to find the same change in other immune and infectious disease, and cannot act as special diagnostic indexes for KD.

The pathogenesis of KD have not been clear so far. However, studies have demonstrated that both immune cells and immune-related genes are involved in the pathogenesis of KD (Sánchez-Manubens et al., 2014; Sakurai et al., 2019). Evaluating the difference of immune cell composition in KD from the perspective of the immune system may have great value in elucidating the molecular mechanism and developing new diagnostic algorithms of KD. Bioinformatics has now been widely used in revealing the molecular mechanism of diseases. CIBERSORTx is an analysis tool to infer the expression of immune cells and obtain various immune cell proportions from samples based on gene expression or sequencing data (Newman et al., 2019).

In this study, we downloaded the microarray datasets of KD from Gene Expression Omnibus (GEO) database and performed differential expression immune-related gene analysis; least absolute shrinkage and selection operator (Lasso) regression algorithm and support vector machine-recursive feature elimination (SVM-RFE) algorithm were used to further screen the diagnostic markers of KD. Based on the diagnostic markers, we have developed and verified a diagnostic model. Subsequently, CIBERSORTx was used to assess the relative content of 22 kinds of immune cell subsets in whole blood in children with KD. In addition, the relationship between diagnostic markers and immune cells was analyzed to better understand the molecular immune mechanism of KD.

## Materials and Methods

### Microarray Data

The raw data of gene chip of GSE73461, GSE68004, GSE18606, GSE73463, and GSE63881 were downloaded from the GEO (https://www.ncbi.nlm.nih.gov/geo/) database. GSE73461 contains 459 samples (all children <17 years of age), including acute KD (*n* = 78), healthy (*n* = 55), bacterial infection (*n* = 52), viral infection (*n* = 94), juvenile idiopathic arthritis (*n* = 66), Henoch-Schönlein purpura (*n* = 18), and infections of uncertain bacterial or viral etiology (*n* = 96). GSE68004 contains 162 samples, and its raw data were divided into two parts, part 1 including acute KD (*n* = 62), healthy (*n* = 14), adenovirus (*n* = 9), and group A *streptococcus* (*n* = 16), and part 2 including acute KD (*n* = 27), healthy (*n* = 23), adenovirus (*n* = 10), and group A *streptococcus* (*n* = 1). GSE18606 contains 48 samples, including acute KD (*n* = 20) and healthy (*n* = 9). GSE73463 contains 233 samples, namely, acute KD (*n* = 146) and convalescent KD (*n* = 87). GSE63881 contains 341 samples, namely, acute KD (*n* = 171) and convalescent KD (*n* = 170). The GSE73461 was used as the training set; GSE68004, GSE18606, GSE73463, and GSE63881 were used as the independent validation set.

### Data Preprocessing and Identification of Differentially Expressed Genes (DEGs)

Data analysis used R software (version 4.0.2, https://www.r-project.org/) and bioconductor packages (http://www.bioconductor.org/). Raw data of GSE73461, GSE68004, GSE18606, GSE73463, and GSE63881 datasets were read, background correction and data normalization through the “limma” package (http://www.bioconductor.org/packages/release/bioc/html/limma.html). Considering the possibility of information loss during the removing batch difference processing, the datasets have not been merged. Linear models of “limma” package was used to screen DEGs of GSE73461 by comparing the expression values between KD and non-KD, and |log2FC| > 1 and adjusted *p* < 0.01 were considered statistically significant. The pheatmap (https://bioconductor.org/packages/release/bioc/html/ heatmaps. html) and ggplot2 (https://cran.r-project.org/web/packages/ggplot2/index.html) packages were used to draw a heatmap and volcano map to show the differential expression of DEGs.

Immune-related genes were downloaded from the Immunology Database and Analysis Portal database (ImmPort, https://www.immport.org/shared/genelists), in which 1,509 genes contain 17 immune categories based on molecular function (Bhattacharya S et al., 2014). The immune-related DEGs were obtained by the DEGs and immune-related genes were overlapped.

### Functional Analysis of Immune-Related Differentially Expressed Genes

To assess the potential biologic functions of immune-related DEGs, Disease Ontology (DO), Gene Ontology (GO), and Kyoto Encyclopedia of Genes and Genomes (KEGG) enrichment analysis were performed by the “clusterProfiler” package (Yu G et al., 2012), respectively. Gene set enrichment analysis (GSEA) was performed on the all immune-related genes expression matrix by the “clusterProfiler” package. *p* < 0.05 was considered statistically significant.

### Screening and Verification of Diagnostic Markers

Based on immune-related DEGs, candidate diagnostic markers for KD were selected through integrated analysis of two algorithms including Lasso logistic regression (Tibshirani R et al., 1996) with penalty conducted by 10-fold cross-validation and SVM-RFE (Huang M et al., 2014). A logistic regression model was used to construct a diagnostic signature based on the candidate immune-related genes. Expression matrices of the GSE68004, GSE18606, GSE73463, and GSE63881 were used as independent datasets to verify each candidate gene and the diagnostic model. Receiver operating characteristic (ROC) curves were used to evaluate the accuracy and efficiency of the candidate genes and the diagnostic model.

### Evaluation of Immune Cell Level

CIBERSORTx (Newman AM et al., 2019) algorithm was used to quantify the proportions of immune cells in the samples of GSE73461. This algorithm contains 22 kinds of immune cells. Barplot and violin diagrams were drawn to visualize the differences of immune cell in different diagnostic samples; correlation heatmap was drawn to visualize the correlation of immune cells.

### Correlation Analysis Between Diagnostic Markers and Immune Cells

Spearman correlation analysis on diagnostic markers and immune cells was performed, and the analysis results were visualized.

### Statistical Analysis

Statistical analyses were completed using the SPSS 22.0 software for Windows (SPSS, Chicago). All values are shown as mean ± standard deviation, or number and percentage (*n*, %). To compare the differences between groups, Student’s *t*-tests were used for Continuity variables. Chi-square test was used to compare frequencies between groups. A two-tailed *p* value < 0.05 was used as a threshold for determining statistical significance.

## Results

### Demographic Features and Differentially Expressed Genes Screening in the Training Set

**FIGURE 1:**
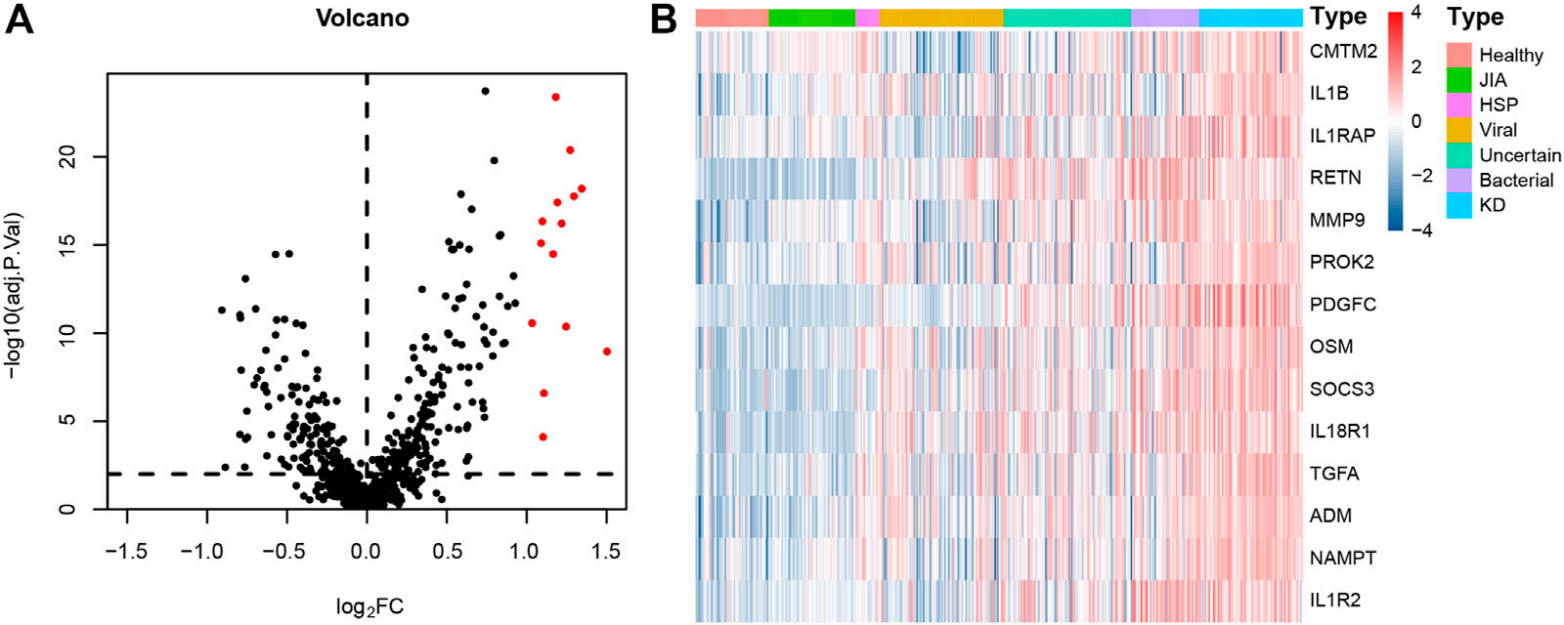
Immune-related DEGs expression volcano plot and heatmap between KD and non-KD. **(A)** Volcano plot of immune-related DEGs; red represents upregulated differential genes, and black represents no significant difference genes (|log2FC| > 1 and adjusted *p* < 0.01). **(B)** Heatmap of immune-related DEGs in KD and non-KD. From blue to red represents the change from low expression to high expression (all immune-related DEGs).

The numbers of children in each diagnostic category and demographic features are summarized in Table 1. A total of 100 DEGs were identified between 78 KD and 381 non-KD samples in the training set with the cutoff criteria of |log_2_FC| > 1 and adjusted *p* < 0.01. Fourteen immune-related DEGs were obtained by intersections with immune-related genes, as shown in the volcano plot and heatmap (Figure 1). All the screened genes were upregulated.

**TABLE 1:** Demographic features of each dataset.

Dataset	Diagnosis	Age (years)			Gender			
		Age	*F*	*p* ^a^	Male	Female	*χ* ^*2*^	*p* ^a^
GSE73461	KD	<17	—	—	43 (9.37%)	35 (7.63%)	0.016	0.900
	Bacterial infection	<17	—	—	22 (4.79%)	30 (6.54)	—	—
	Viral infection	<17	—	—	66 (14.38%)	28 (6.10%)	—	—
	Juvenile idiopathic arthritis (JIA)	<17	—	—	25 (5.45%)	41 (8.93%)	—	—
	Henoch-Schönlein purpura (HSP)	<17	—	—	9 (1.96%)	9 (1.96%)	—	—
	Uncertain	<17	—	—	62 (13.51%)	34 (7.41%)	—	—
	Healthy	<17	—	—	29 (6.32%)	26 (5.66%)	—	—
GSE68004	Complete KD	3.68 ± 2.50	15.889	<0.001	32 (19.75%)	44 (27.16%)	0.103	0.748
	Incomplete KD	4.65 ± 3.52	—	—	9 (5.56%)	4 (2.47)	—	—
	Adenovirus (HAdV)	4.14 ± 2.48	—	—	10 (6.17%)	9 (5.56%)	—	—
	Group A streptococcal disease (GAS)	5.46 ± 4.12	—	—	10 (6.17%)	7 (4.32%)	—	—
	Healthy	7.14 ± 4.62	—	—	15 (9.26%)	22 (13.58%)	—	—
GSE18606	KD	2.98 ± 2.13	1.121	0.299	10 (34.48%)	10 (34.48%)	0.697	0.404
	Healthy	2.11 ± 1.46	—	—	3 (10.34%)	6 (20.69%)	—	—
GSE73463	Acute KD	<17	—	—	60 (25.75%)	86 (36.91%)	0.684	0.408
	Convalescent KD	<17	—	—	31 (13.30%)	56 (24.03%)	—	—
GSE63881	Acute KD	3.32 ± 2.83	0.001	0.975	69 (20.23%)	102 (29.91%)	0.002	0.964
	Convalescent KD	3.32 ± 2.84	—	—	69 (20.23%)	101 (29.62%)	—	—

a Comparison between the KD group and the non-KD group, or between the acute KD group and the convalescent KD group.

### Functional Correlation Analysis of Immune-Related Differentially Expressed Genes

**FIGURE 2:**
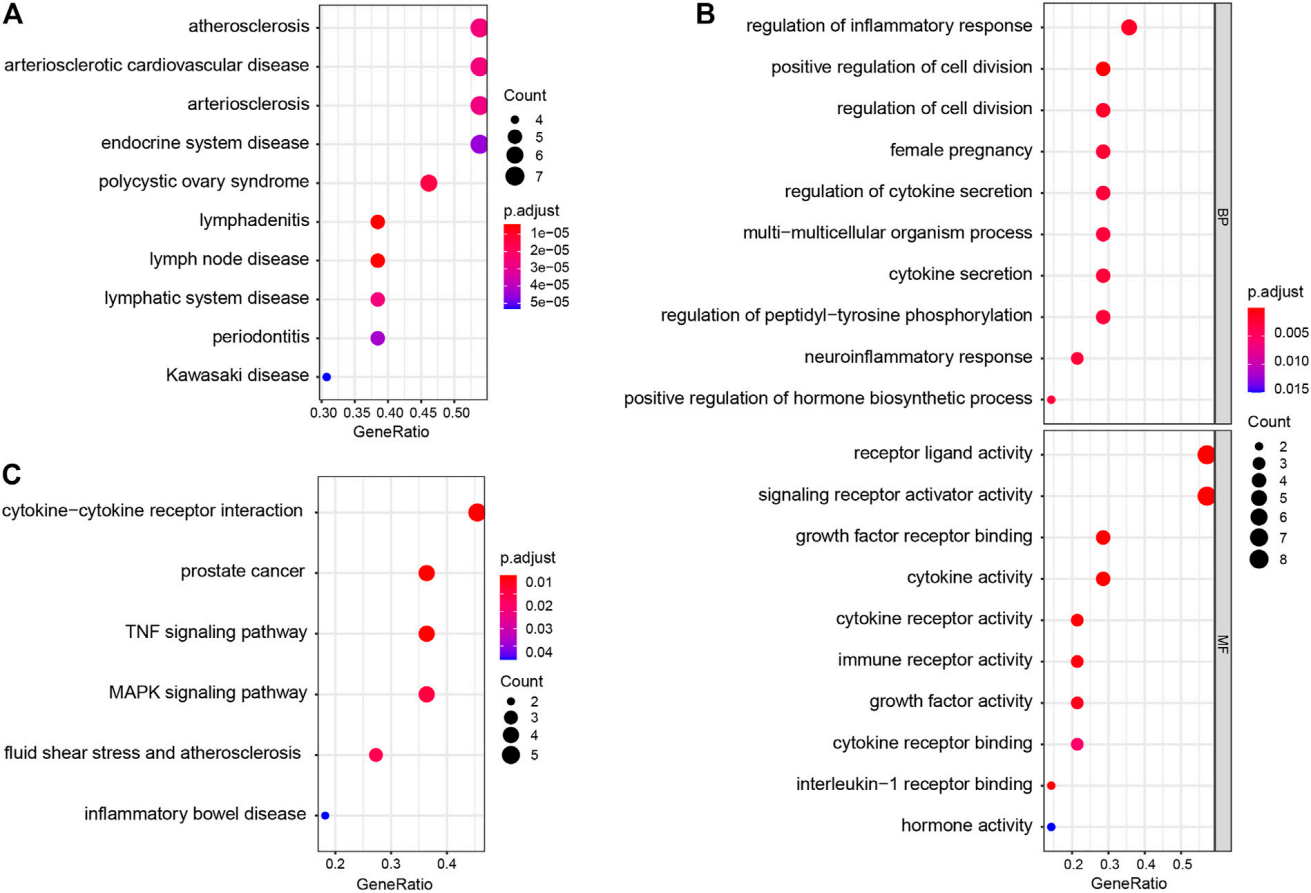
Disease Ontology (DO), Gene Ontology (GO), and Kyoto Encyclopedia of Genes and Genomes (KEGG) enrichment analysis of immune-related DEGs. **(A)** DO enrichment analysis (top 10 according to adjusted *p* value). **(B)** GO enrichment analysis; the figure represents biological process and molecular function (top 10 according to adjusted *p* value, respectively). **(C)** KEGG pathway analysis results of immune-related DEGs.

**FIGURE 3:**
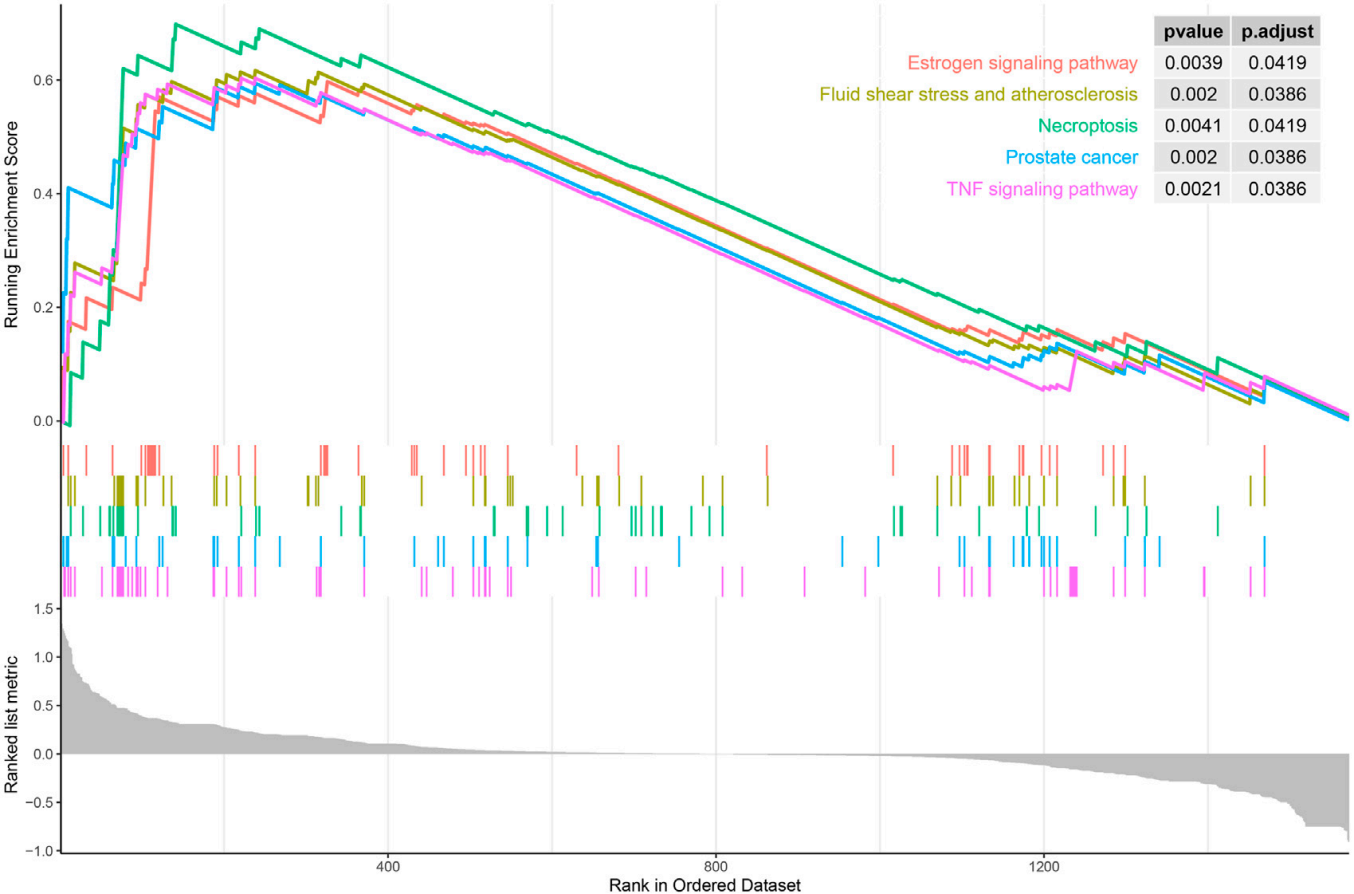
Gene set enrichment analysis (GSEA) of all immune-related genes in the training set (top five according to normalized enrichment score).

DO enrichment analysis showed that the immune-related DEGs were mainly involved atherosclerosis, arteriosclerotic cardiovascular disease, arteriosclerosis, endocrine system disease, polycystic ovary syndrome, lymphadenitis, lymph node disease, lymphatic system disease, periodontitis, and Kawasaki disease (Figure 2A). GO analysis revealed that mainly functional categories in the biological processes (BP) involved regulation of inflammatory response, positive regulation of cell division, regulation of cell division, female pregnancy, regulation of cytokine secretion, multi-multicellular organism process, cytokine secretion, regulation of peptidyl-tyrosine phosphorylation, neuroinflammatory response, and positive regulation of hormone biosynthetic process; for molecular function (MF), the main terms involved receptor ligand activity, signaling receptor activator activity, growth factor receptor binding, cytokine activity, cytokine receptor activity, immune receptor activity, growth factor activity, cytokine receptor binding, interleukin-1 receptor binding, and hormone activity; for cellular components (CC), no term was enriched at the same cutoff criteria of BP and MF (Figure 2B). KEGG pathway analysis revealed that the immune-related DEGs mainly involved cytokine–cytokine receptor interaction, prostate cancer, TNF signaling pathway, MAPK signaling pathway, fluid shear stress and atherosclerosis, and inflammatory bowel disease (Figure 2C). GSEA analysis showed that the enriched pathways mainly involved TNF signaling pathway, prostate cancer, necroptosis, fluid shear stress and atherosclerosis, estrogen signaling pathway, IL-17 signaling pathway, NOD-like receptor signaling pathway, neuroactive ligand–receptor interaction, osteoclast differentiation, and Toll-like receptor signaling pathway (Figure 3).

### Screening and Verification of Diagnostic Markers

**FIGURE 4:**
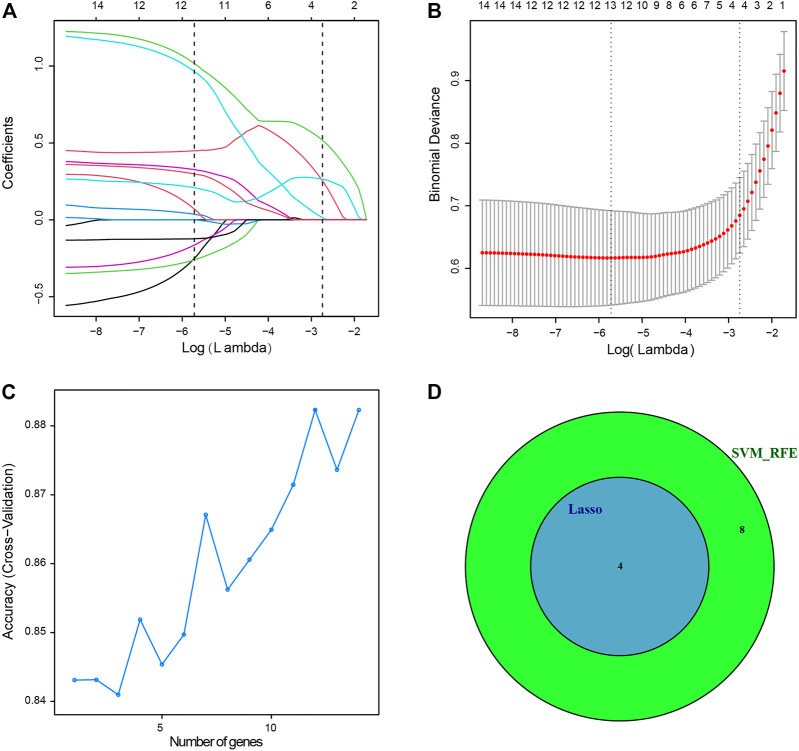
Two algorithms were used for diagnostic markers for selection. **(A)** Least absolute shrinkage and selection operator (Lasso) algorithm to screen diagnostic markers, 10-fold cross-validation for selection in the Lasso model. Different colors represent different genes. **(B)** Lasso coefficient profiles of 14 immune-related DEGs. **(C)** The accuracy of the estimate generation for the support vector machine-recursive feature elimination (SVM-RFE) algorithm. **(D)** The individual feature selection by Lasso and SVM-RFE algorithms and the intersection of diagnostic markers obtained by the two algorithms.

**FIGURE 5:**
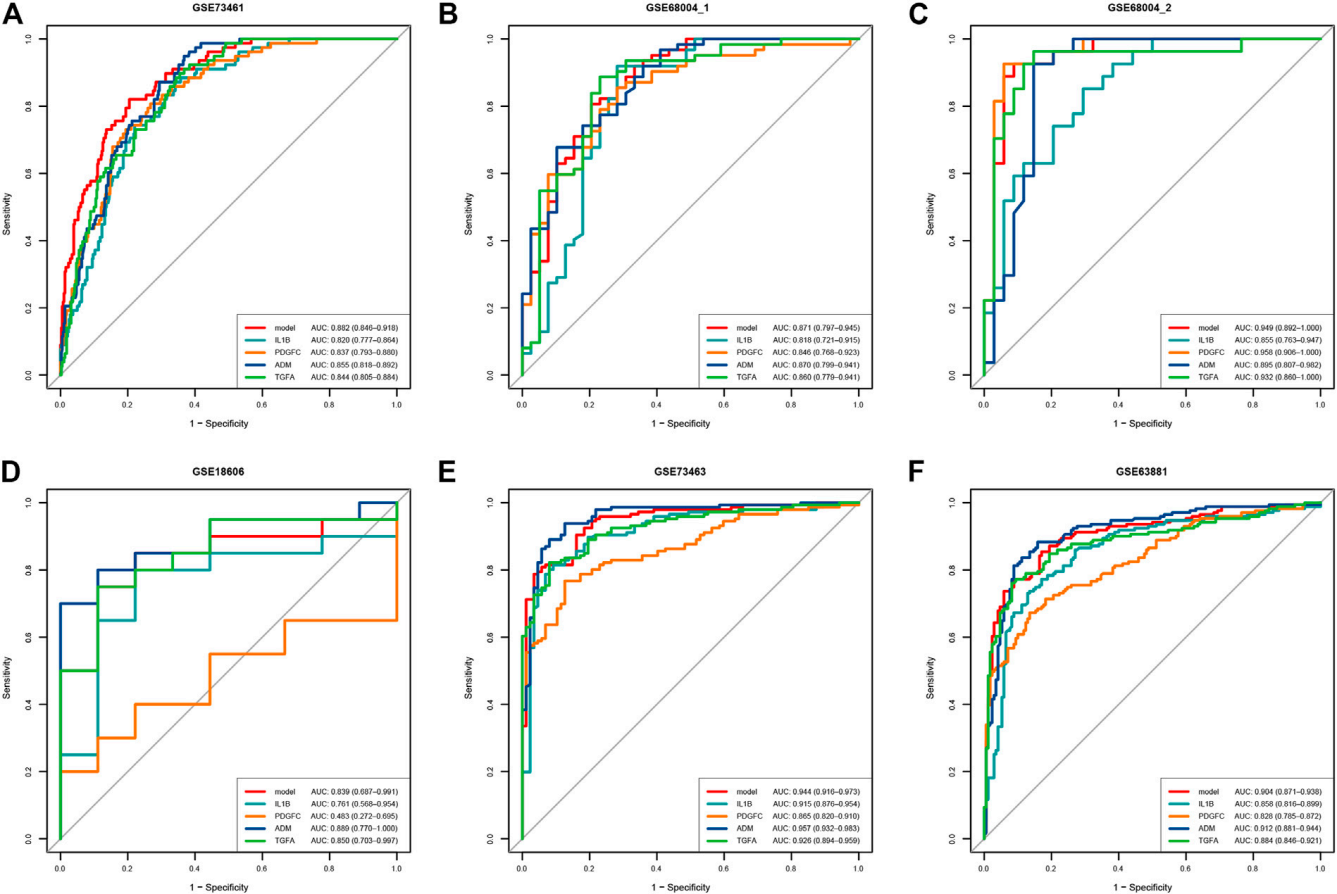
ROC curve of the predictive efficacy verification of the single diagnostic marker and 4-gene diagnostic model. **(A)** ROC curve of training set. **(B–D)** ROC curve of verification sets, each dataset contains acute KD and non-KD samples. **(E,F)** ROC curve of verification sets, each dataset contains acute KD and convalescent KD samples.

Four genes were identified from immune-related DEGs by using the Lasso algorithm (Figures 4A,B), and twelve genes were selected by using the SVM-RFE algorithm (Figure 4C). After overlapping the gene markers obtained by the two algorithms, four candidate diagnosis-related genes were obtained (Figure 4D). In order to further test the diagnostic efficacy of the four candidate genes, a logistic regression model was established (Table 2), and validation was performed through the GSE68004, GSE18606, GSE73463, and GSE63881 datasets. The area under the curve (AUC) of ROC for the logistic regression model was 0.882 in the training set (Figure 5A), and reached a high level in all validation sets (Figures 5B–F).

**TABLE 2:** Diagnostic markers for KD.

Intercept and genes	*β*	Odds ratio (95% CI)	*p*
Intercept	−23.5334	—	—
*IL1B*	0.7655	2.150 (1.192–4.019)	0.013
*ADM*	0.6158	1.851 (0.785–4.555)	0.169
*PDGFC*	0.7697	2.159 (1.503–3.161)	<0.001
*TGFA*	0.2468	1.280 (0.736–2.238)	0.383

### Immune Cell Analyses Results

**FIGURE 6:**
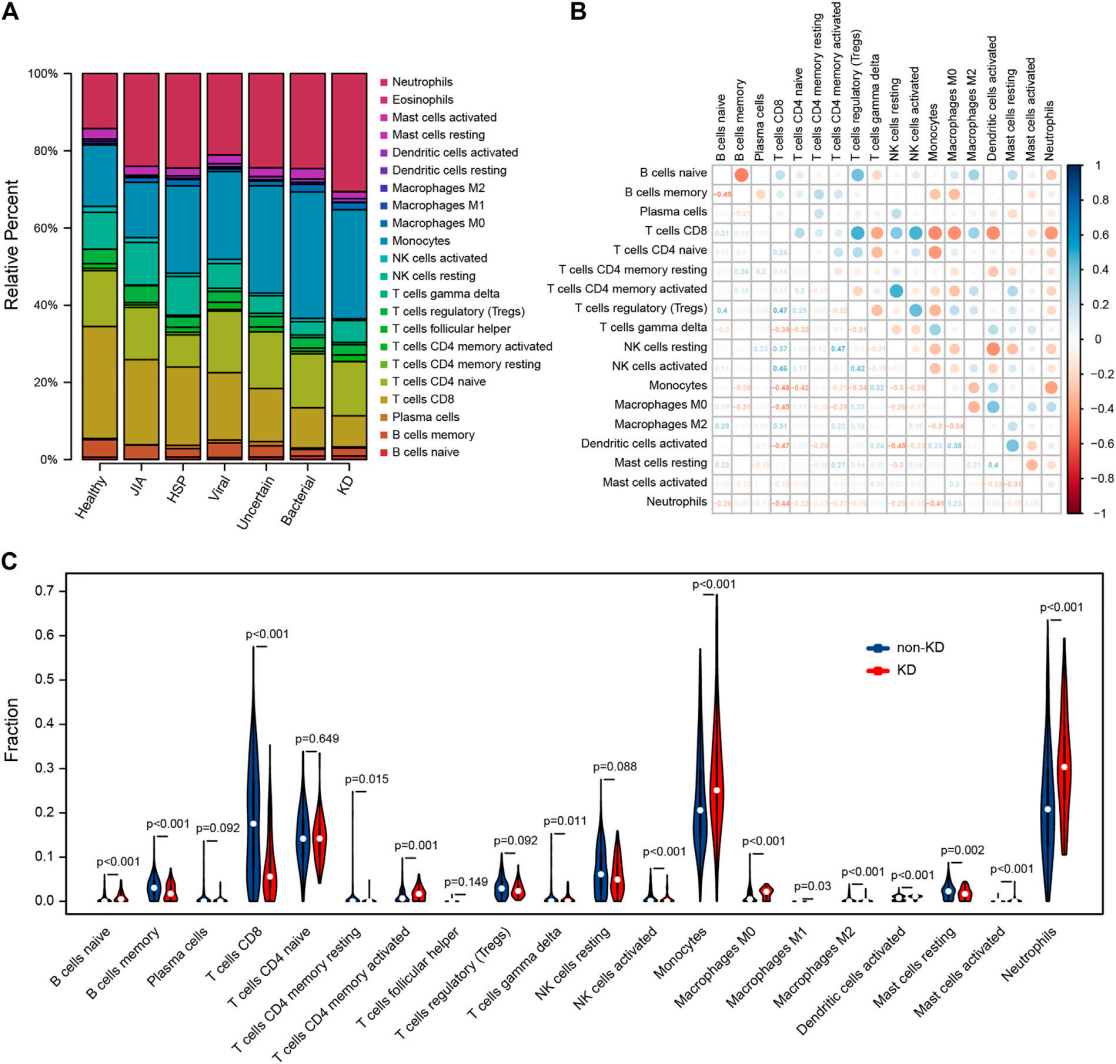
Evaluation and visualization of immune cell analyses. **(A)** Relative percentage of 22 immune cell subpopulations in 459 samples from GSE73461 dataset, results from different diagnostic classifications were averaged. **(B)** Correlation plot of 18 immune cells (4 types of immune cells had an estimated abundance of 0) in 78 KD samples from GSE73461. The size of the colored dots represents the strength of the correlation; blue indicates the positive correlation, and red indicates the negative correlation; the larger the dots and the darker the color, the stronger the correlation. **(C)** The difference of 20 types of immune cells between KD and non-KD (2 types of immune cells had an estimated abundance of 0); blue represents non-KD, and red represents KD; *p* < 0.05 was considered statistically significant.

Using CIBERSORTx algorithm, we first investigated the differences of 22 immune cell subsets between KD and non-KD whole blood samples; results from different diagnostic classifications were averaged (Figure 6A). The correlation plot of 18 immune cells (4 types of immune cells had an estimated abundance of 0) in training set KD samples showed that CD8 T cells had a significant low positive correlation with resting NK cells (*r* = 0.426, *p* = 0.001), a significant low negative correlation with macrophages M0 (*r* = −0.378, *p* < 0.001), and a significant moderate negative correlation with monocytes, activated dendritic cells, and neutrophils (*r* = −0.561, −0.591, and −0.624; all *p* < 0.001). Regulatory T cells (Tregs) had a significant low negative correlation with monocytes (*r* = −0.318, *p* = 0.003). Resting NK cells had a low negative correlation with monocytes and activated dendritic cells (*r* = −0.462 and −0.432, *p* = 0.008 and <0.001). Activated dendritic cells had a low positive correlation with monocytes and resting mast cells (*r* = −0.309 and 0.386, *p* = 0.042 and <0.001) (Figure 6B). Compared with non-KD, whole blood of KD patients contained a higher proportion of naïve B cells, activated memory CD4 T cells, gamma delta T cells, monocytes, M0 macrophages, activated dendritic cells, activated mast cells, and neutrophils, while the proportions of memory B cells, CD8 T cells, activated memory CD4 T cells, activated NK cells, M1 and M2 macrophages, and resting mast cells were relatively lower (Figure 6C).

### Correlation Analysis Between Diagnostic Markers and Immune Cells in Kawasaki Disease

**FIGURE 7:**
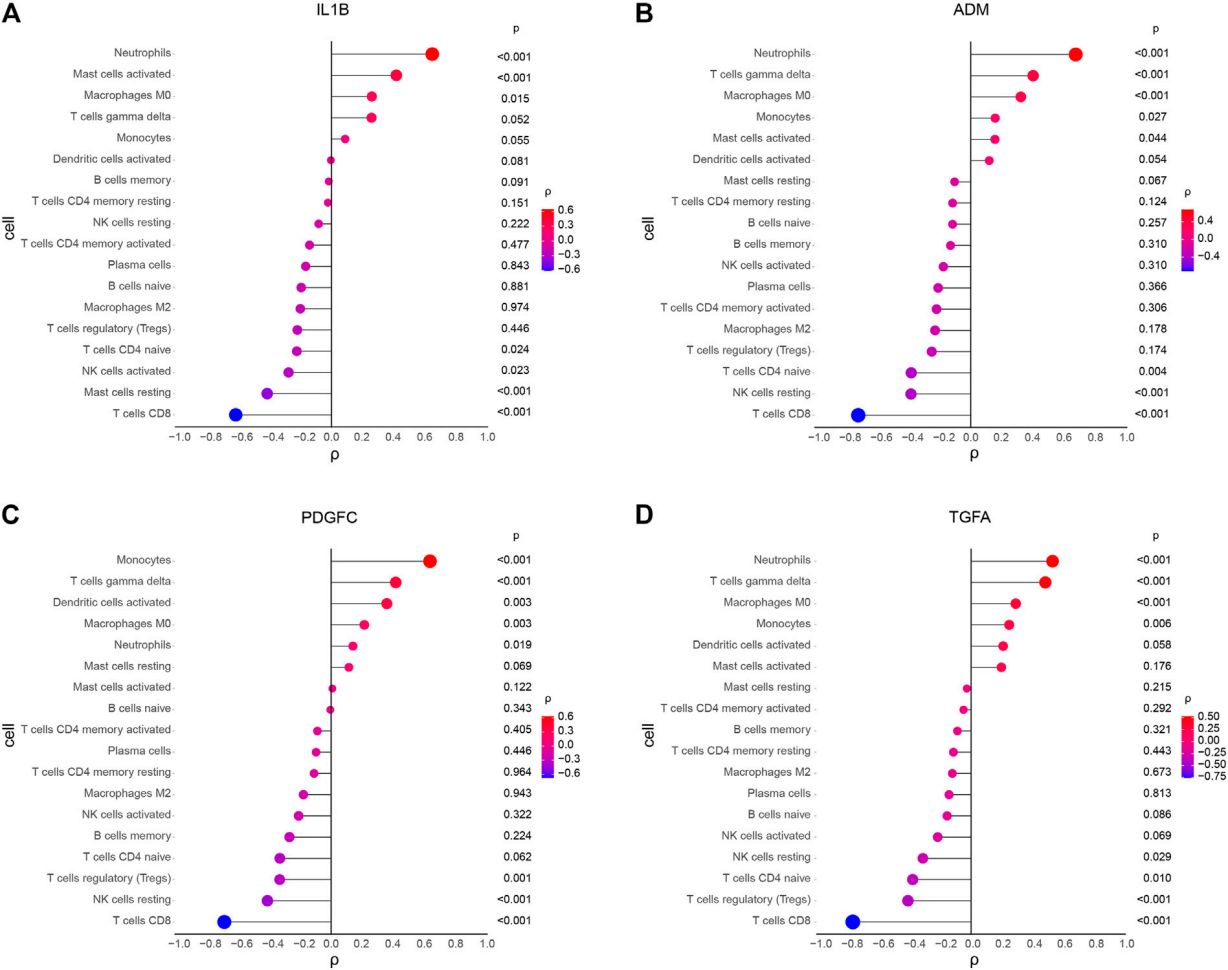
Spearman correlation analysis between diagnostic markers and immune cells in KD. **(A)** Correlation between IL1B and immune cells. **(B)** Correlation between ADM and immune cells. **(C)** Correlation between PDGFC and immune cells. **(D)** Correlation between TGFA and immune cells. The size and color of the dots represents the *p*-value and the strength of the correlation between diagnostic markers and immune cells; red represents the positive correlation, and blue represents the negative correlation. The larger the dots, the lower the *p*-value, and the bluer or redder, the stronger the correlation. *p* < 0.05 was considered statistically significant.

Correlation analysis showed that *IL1B* was positively correlated with neutrophils, activated mast cells, and M0 macrophages, and negatively correlated with CD8 T cells, resting mast cells, activated NK cells, and naive CD4 T cells (Figure 7A). *ADM* was positively correlated with neutrophils, gamma delta T cells, M0 macrophages, monocytes, and activated mast cells, and negatively correlated with CD8 T cells, resting NK cells, and naïve CD4 T cells (Figure 7B). *PDGFC* was positively correlated with monocytes, gamma delta T cells, activated dendritic cells, M0 macrophages, and neutrophils, and negatively correlated with CD8 T cells, resting NK cells, and regulatory T cells (Tregs) (Figure 7C). *TGFA* was positively correlated with neutrophils, gamma delta T cells, M0 macrophages, and monocytes, and negatively correlated with CD8 T cells, regulatory T cells (Tregs), naive CD4 T cells, and resting NK cells (Figure 7D).

## Discussion

The diagnostic criterion of KD mainly depends on clinical symptoms and signs, but some patients, especially infants under 6 months old and adolescents, did not meet the criterion in the early stage of disease, and thus it is difficult to make a timely diagnosis. Delayed diagnosis and treatment may cause serious adverse outcomes such as CAAs. Abnormal gene expression level has been studied in diagnosis of KD (Kuo et al., 2016; Jaggi et al., 2018; Wright et al., 2018). However, these studies proposed that biomarkers were too many or not further validated by the external cohort. In this study, we developed a simple and concise diagnosis model, composed of four genes, by analyzing expression levels of immune-related genes. Verification of this model was performed by three other external datasets including KD and non-KD, and shows a great capacity for diagnosis of KD. At the same time, this model was validated with two datasets including acute KD and convalescent KD.

The training set was used to identify 14 immune-related DEGs. DO enrichment analysis showed that the DEGs were mainly related to KD, atherosclerosis, arteriosclerosis, and arteriosclerotic cardiovascular disease. This enrichment result is consistent with previous studies that have shown an increased risk of early atherosclerosis in children with KD (Cho et al., 2014; Zhang et al., 2016). In addition, both KEGG and GSEA were enriched in fluid shear stress and atherosclerosis, and tumor necrosis factor (TNF) signaling pathway. Masaki et al. (Xie et al., 2018). revealed that gene polymorphisms of TNF may affect KD susceptibility, and TNF-*α* is associated with the development of CALs in KD patients (Hu et al., 2017; Guo et al., 2020). The results indicated that the analysis results of this study are accurate. The etiology of KD is considered to have an infectious trigger in a genetically susceptible individual, followed by the activation of the immune system (Yim et al., 2013). The main trigger pathogens suspected were the viral agents (Usta Guc et al., 2008; Alexoudi et al., 2011; Chang et al., 2014; Shulman et al., 2015; Kim et al., 2016); some studies have linked triggers to bacteria agents (Galeotti et al., 2010; Alexoudi et al., 2011). However, in our study, the levels of immune-related differentially expressed genes (DEGs) in bacterial infection samples were more similar to those of KD than viral infection samples. It is speculated that the bacteria triggering may be the main factor of the activation of the KD immune system.

Based on immune-related DEGs and machine learning algorithms, Lasso and SVM-RFE were performed to screen KD diagnostic markers. By integrating the features of the two algorithms, *IL1B*, *ADM*, *PDGFC*, and *TGFA* were selected as diagnostic biomarkers. Although the single biomarker for diagnosis and prognosis has been widely reported, value and robustness are also a major concern. So, we further developed a logistics prediction model with these 4 markers, and further external verification shows that the model was reliable. In our validation sets, two sets only contain acute KD and convalescent KD, but both diagnostic markers and model showed well differentiation. Interleukin-1*β* (IL-1*β*) is a potential inflammation-causing cytokine released predominantly by immune-derived cells. Studies indicate that IL-1*β* has a significant role in several aspects of vascular inflammation (Chamberlain et al., 2006); there is a high level of IL-1*β* in the endothelium of atherosclerotic coronary arteries (Chibana et al., 2017) and might be one of the targets of atherosclerotic protective therapy (Ridker et al., 2017). Similarly, there are reports that the gene polymorphisms of IL-1*β* may be associated with initial IVIG treatment failure (Weng et al., 2010) and significantly impact the risk of CAL formation in children with KD (Fu et al., 2019). IL-1*β* may also increase the production of neutrophil extracellular traps by activating neutrophils, thereby inducing the progression of coronary artery ectasia (Guo et al., 2020). Adrenomedullin (*ADM*) is a vasoactive peptide involved in vasodilation and regulation of endothelial function. ADM inhibits the apoptosis of cultured endothelial cells and the high permeability of human vascular smooth muscle cells induced by agonists (Hippenstiel et al., 2002). Plasma ADM levels can reflect the degree of endothelial damage in atherosclerosis patients (Bunton et al., 2004). It is a good indicator of prognosis in patients with coronary artery disease (Wong et al., 2012). In KD, the expression level of *ADM* gene in plasma has been reported to be elevated (Nishida et al., 2001; Nomura et al., 2005); plasma ADM levels were higher in KD patients developed CAAs than in those did not (Nishida K et al., 2001). Platelet-derived growth factors (PDGFs) play an important role in vascular pathologies such as atherosclerosis, restenosis, and aortic aneurysm. PDGF-C can promote the migration and proliferation of macrophages (Boor et al., 2010), endothelial cells (Li et al., 2005), and vascular smooth muscle cells (Crawford et al., 2009). Karvinen et al. (2009) have revealed that PDGF-C is expressed in different stages of atherosclerosis. However, as a new member of the PDGF family, PDGF-C has been less studied in cardiovascular diseases since its discovery. However, *PDGFC* failed in GSE18606 when independently distinguishing KD from non-KD, which may be related to the small sample size of this dataset. Transforming growth factor *α* (TGF-*α*) as a member of the epidermal growth factor (EGF) family has been associated with cerebrovascular diseases (Dai et al., 2020) and pulmonary vascular disease (Le Cras et al., 2003; Bouzina et al., 2018). Similarly, TGF-*α* has been less studied in cardiovascular disease.

CIBERSORTx evaluation showed that the abundance of naive B cells, activated memory CD4 T cells, gamma delta T cells, monocytes, M0 macrophages, activated dendritic cells, activated mast cells, and neutrophils was increased, and the abundance of memory B cells, CD8 T cells, activated memory CD4 T cells, activated NK cells, M1 and M2 macrophages, and resting mast cells was decreased. Studies have shown that, CD8 T cells, neutrophils, IgA plasma cells, and macrophages infiltrate and destroy the internal elastic lamina of medium-sized vessels (Takahashi et al., 2005; Alexoudi et al., 2011; Orenstein et al., 2012). However, compared to CD8 T cells, CD4 T cells predominate in peripheral blood (Alexoudi et al., 2011), which may be due to the CD8 T-cell transmission to tissues during the development of KD. In the acute phase of KD, the increase in the number of circulating neutrophils and activation eventually cause endothelial dysfunction (Sakurai et al., 2019). In addition, our results revealed that CD8 T cells are closely related to resting NK cells, macrophages M0, monocytes, activated dendritic cells, and neutrophils; regulatory T cells (Tregs) are closely related to monocytes; resting NK cells are closely related to monocytes and activated dendritic cells; and activated dendritic cells are closely related to monocytes and resting mast cells. Considering the complexity of the immune system, it is difficult to study the immune cells of KD. Our analysis provides reference for the study of immune response in the pathogenesis of KD.

We used novel scientific algorithms to identify the diagnostic markers for KD and verified them with multiple external datasets. CIBERSORTx was used to analyze the proportion of various immune cells in KD whole blood for the first time. Although the diagnostic model in this study is composed of only four genes with significant diagnostic impact, which makes it handy to use in clinical practice, it still has some limitations. Firstly, our research is the second mining and analysis of previously published datasets, and the sample size of two validation sets is small. Secondly, CIBERSORTx analysis is based on limited genetic data to infer cell-type abundance and cell-type-specific gene expression, which may deviate from the actual situation. In addition, the reliability of the results of our study needs further experiments to verify.

## Conclusion

In the present study, 4 immune-related DEGs were identified, and a diagnostic model for KD was established and verified. The biological functions and pathways of the immune-related genes provide a detailed molecular mechanism for understanding the pathogenesis of KD. By CIBERSORTx algorithms, we found a difference in immune cells between KD and non-KD. These immune cells may play a key role in the pathogenesis and development of KD. Further analysis of the relation between immune-related DEGs and immune cells will be helpful to further explore the pathogenesis of KD and may determine the immunotherapy targets of KD (Bajolle et al., 2014).

## Data Availability

Publicly available datasets were analyzed in this study. These data can be found here: https://www.ncbi.nlm.nih.gov/gds/ (Gene Expression Omnibus) GSE73461, GSE68004, GSE18606, GSE73463, and GSE63881.
